# Unsuspected Pneumocystis pneumonia in an HIV-seronegative patient with untreated lung cancer: circa case report

**DOI:** 10.1186/1752-1947-1-115

**Published:** 2007-10-28

**Authors:** Cai Chuang, Xie Zhanhong, Gu Yinyin, Zeng Qingsi, Zhong Shuqing, Zhong Nanshan

**Affiliations:** 1 Guangzhou Institute of Respiratory Disease, First Affiliated Hospital of Guangzhou Medical University, Guangzhou 510012, China.

## Abstract

**Background:**

Patients with solid malignant tumours are at increased risk of Pneumocystis *jiroveci *infection from immunosuppression as a result of chemotherapy and/or radiotherapy, but active Pneumocystis pneumonia (PCP) in untreated lung cancer is uncommon.

**Case presentation:**

A 43-year-old woman presented with prolonged fever, progressive dyspnoea, diffuse alveolar and interstitial infiltrates. Malignant cells were found on sputum cytology, confirming the diagnosis of lung cancer. She had been treated with corticosteroids and antibiotics but did not receive chemotherapy or radiotherapy. Pneumocystis *jiroveci *was later found in the sputum but she proved to be HIV negative.

**Conclusion:**

Unsuspected PCP can occur in chemotherapy and radiotherapy-naïve, HIV-seronegative patients with lung cancer. The complex clinicoradiological manifestations of PCP with underlying lung cancer can lead to delay in diagnosis and may worsen the prognosis.

## Background

Pneumocystis *jiroveci *pneumonia (PCP, formerly known as Pneumocystis *carinii *pneumonia) has been increasingly reported as a severe opportunistic infection in HIV-seronegative patients with solid tumours (brain, lung, breast and ovarian cancer), as a sequel to severe immunosuppression from chemotherapy and/or radiotherapy with or without corticosteroids [1-5].

We present a case of unsuspected PCP in an HIV-seronegative patient with untreated lung cancer, manifested as persistent fever, progressive dyspnoea, and diffuse alveolar and interstitial infiltrates.

## Case presentation

A 43-year-old nonsmoking female was referred to our hospital for persistent fever, progressive dyspnoea, and diffuse alveolar and interstitial infiltrates. The patient complained of intermittent mild to moderate fever, progressive dyspnoea, increasing exportation and paroxysmal wheezing for 6 weeks. Her past history was unremarkable, without known exposure to occupational or environmental hazards. She was initially diagnosed as community acquired pneumonia in her local hospital, treated with intravenous levofloxacin, which was escalated to cephatriaxone when her symptoms worsened. Intravenous dexamethasone (10 mg to 20 mg prn) with aminophylline was administered irregularly to relieve her dyspnoea and wheezing. She developed orthopnoea with high fever and copious gel-like phlegm four weeks after the onset of symptoms, so she was transferred to our hospital.

On admission, she displayed orthopnoea, cyanosis, finger clubbing and nonpitting edoema in the lower extremities. Vital signs: body temperature 38.8°C, HR 124 beats/min, respiratory rate 32 breath/min, and Bp 124/76 mmHg. Fine crackles were audible at both bases with resonant wheezing throughout inspiration and expiration. Superficial lymph nodes, heart and abdomen were unremarkable.

Laboratory workup: complete blood count revealed WBC elevation with WBC 15.4 ×10^9^/L, granulocytes 86%, lymphocytes 14%, RBC and PLT were in normal range. Serum liver and renal biochemistry as well as electrolytes were unremarkable. Serum CEA 13.4 μg/L (0–5 μg/L), D-dimer 680 ng/mL (< 200 ng/mL). Arterial blood gas analysis (nasal oxygen, 3 L/min): pH 7.42, PaO_2_6.54 kPa, PaCO_2 _3.86 kPa.

**Figure 1:**
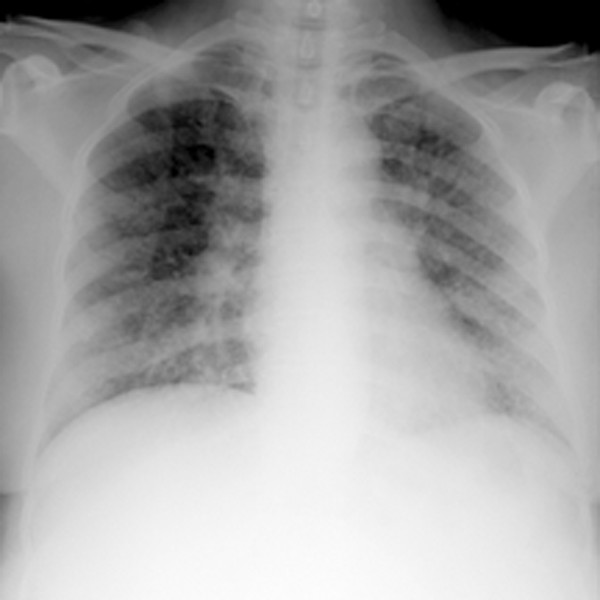
Chest radiograph showing diffuse nodular and patchy pulmonary infiltrates, and right hilar widening.

**Figure 2:**
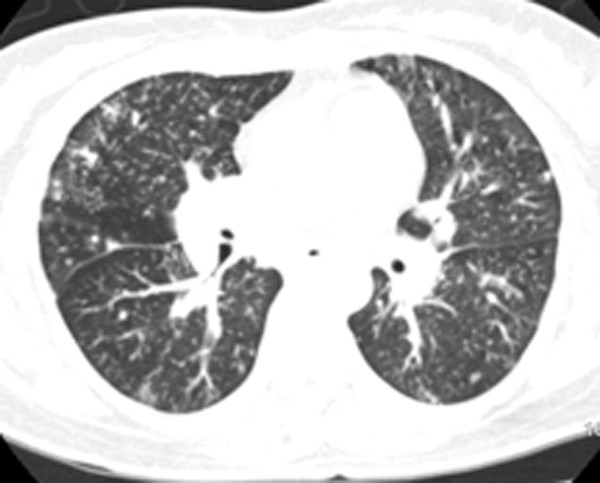
Chest CT displaying diffuse nodular parenchymal infiltrates with partial confluence.

Chest radiograph (Fig. 1) revealed diffuse nodular and patchy consolidations with predominance in the lower lung fields, and right hilar expansion. Chest CT scanning (Fig. 2) demonstrated diffuse alveolar and interstitial infiltrates with partial confluence and adenopathy in both hilar regions. CT angiography was negative for pulmonary embolism.

Diffuse bronchioloalveolar carcinoma (BAC) complicated by acute respiratory failure was suspected, malignancy was confirmed with evidence of malignant cells repeatedly found in sputum smears examined by a lung pathologist (Dr Gu YY). Intubation and invasive mechanical ventilation was strongly recommended because of her critical condition, but was declined by the patient and her family. Non-invasive ventilation (CPAP) was given as an alternative to treat the respiratory failure, with imipenem/cilastin to target the suspected bacterial pneumonia. Her orthopnoea and high fever remained despite treatment, with peripheral WBC elevated to 22.6 × 10^9^/L, granulocytes 95%, lymphocytes 5%, yet repeated blood and sputum cultures for bacteria were negative. Empiric vancomycin was added for possible gram-positive cocci infections to no avail.

**Figure 3:**
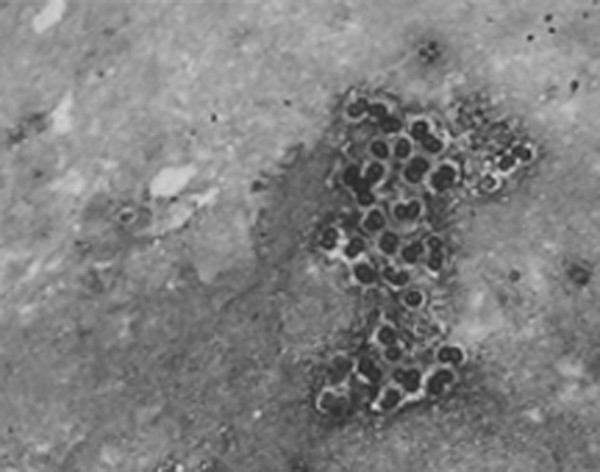
Methamine silver stain demonstrating clusters of Pneumocystis *jiroveci *cysts in the sputum (×100)

Four days after admission, clusters of Pneumocystis *jiroveci *cysts were unexpectedly and repeatedly identified in her sputa using methamine silver stain (Fig. 3). The patient was therefore diagnosed with PCP. Oral TMP-SMX (2 double-strength tab, tid, as intravenous pentamdine or trimethoprim/sulfamethoxazole was not available in Guangzhou), with intravenous methylprednisolone 40 mg qd as adjunctive therapy, was initiated with the original antibiotic regimen still continued. Further investigations found that her peripheral blood CD4 T lymphocyte was 189/μL with CD4/CD8 ratio as 1.4: 1, C reactive protein (CRP) was 26 mg/L (0–8 mg/L), serum lactate dehydrogenase (LDH) was 371 IU/L (35–90 IU/L). She tested negative for HIV and cytomegalovirus. Her family members including her husband and children were also HIV- seronegative. When 7 days' treatment with co-trimoxazole failed to alleviate her respiratory distress and high fever, her family opted to withdraw her from therapy and obtained her discharge. She died of respiratory failure three days later.

## Discussion

The patient presented with progressive dyspnoea, copious glutinous sputum, diffuse alveolar and interstitial infiltrates on chest radiograph and CT. These are consistent with the clinicoradiological features of the diffuse form of BAC [6]. In addition, with the cytological evidence of malignancy, a clinical diagnosis of diffuse form of BAC could be reached. However, as the definitive diagnosis of BAC requires histological evidence of malignancy from resected or biopsied lung tissues, whereas in the present case, invasive procedures such as transbronchial lung biopsy were precluded due to the patient's critical condition and lack of invasive mechanical ventilation to ensure reliable oxygenation. So, for this patient, the appropriate diagnosis is lung cancer, most likely, diffuse form of BAC.

PCP is relatively uncommon in lung cancer, as a sequel to immunosuppression, it had been reported sporadically in lung cancer patients [3,7]. All these patients had undergone chemotherapy or radiotherapy with or without corticosteroids, before the onset of PCP. Recently, Maskell et al revealed asymptomatic colonization of Pneumocystis *jiroveci *in lung cancer patients by fibrobronchoscopy, and de la Horra et al identified 12 cases of unsuspected subclinical Pneumocystis *jiroveci *infection using polymerase chain reaction in fixed lung samples from hospitalized patients died of lung carcinoma untreated by radiotherapy or chemotherapy [8,9]. To our knowledge, the present case is the first to document active unsuspected PCP in an untreated, HIV- seronegative patient with lung cancer.

Though fever, dry cough and progressive dysponea with pulmonary infiltrates of alveolar pattern are typical of PCP, these manifestations are nonspecific. Definitive diagnosis of PCP requires identification of Pneumocystis *jiroveci *in respiratory secretions, bronchial alveolar lavage fluid or biopsied lung tissues [1,10]. When the presentation is complicated by the presence of underlying lung cancer, the diagnosis of PCP becomes more challenging as the clinical picture becomes more atypical and confusing. In the present case, the differential diagnosis for the fever, orthopnoea and diffuse alveolar and interstitial infiltrates, should include other superimposed infections, pulmonary embolism and broncholymphatic dissemination of the neoplasm. Both PCP and advanced lung cancer might have contributed to progressive dyspnoea and refractory respiratory failure, as well as the radiological pattern of infiltrates, but the manifestation of copious glutinous sputum is atypical of PCP, and points to lung cancer, most possibly BAC.

Although this HIV-seronegative patient was radiotherapy and chemotherapy naïve, she displayed impaired cellular immunity with significant reduction of peripheral CD4 T lymphocytes. This lymphocytic subset is believed to play a crucial role in the pathogenesis of PCP by orchestrating host immune response responsible for eradication of Pneumocystis *jiroveci*. Reduction of peripheral CD4 T lymphocytes below 200/μL is associated with a higher incidence of PCP in both AIDS and non-AIDS patients [1,10,11]. The mechanism for CD4 T lymphocyte decline without lymphopenia in this patient was unclear, the preceding exposure to high dose dexamethasone might have been an important contributing factor, since corticosteroids can reduce CD4 T lymphocytes numbers and inhibit their functions [1,2,10], thus an important risk factor for colonization of Pneumocystis *jiroveci *in patients with chronic obstructive pulmonary disease or lung cancer [8]. And chronic steroid therapy in patients with brain tumours, connective collagen diseases, or even in child with asthma, is frequently associated with active PCP, as a complication of severe immunosuppression from prolonged steroid use [1,2,8,12]. In addition, host cellular immunity may be compromised by neoplasms which inhibit the activation of T lymphocytes [13].

Severe cellular immunodeficiency resulting from systemic corticosteroids treatment must have led to activation of latent Pneumocystis *jiroveci *infection in our patient, as recent evidence suggests that approximately 20% of patients with chronic lung diseases including lung cancer are asymptomatic carriers of Pneumocystis *jiroveci *[8], which progressed to severe active PCP following high-dose dexamethasone treatment, since other transmission pathways, such as nosocomial infection or cross-transmission among family members were less likely.

As this case demonstrates, significant increase of LDH and CRP reflecting severe systematic inflammation, and severe hypoxemia requiring mechanical ventilation may be associated with a poor clinical outcome. These parameters have been reported to be prognostic predictors of PCP [1,10,14].

Possible resistance to co-trimoxazole by infective Pneumocystis *jiroveci*, concurrent bacterial infections, progression of advanced carcinoma especially by broncholymphatic dissemination might also have contributed to the treatment failure.

Despite the increasing awareness of active PCP in severely immunosuppressed non-AIDS patients, unsuspected PCP cases are still not uncommon due to an increase in the susceptible population, and the complexities of diagnosis, especially when masked by the manifestations of underlying diseases and their complications. For example, the clinicoradiological manifestations of our patient might have been reasonably and convincingly explained by BAC and concurrent bacterial infections if Pneumocystis *jiroveci *had not been found in the sputum. Similarly, 9 cases of unsuspected active PCP were retrospectively identified in 50 infants with PCP as presenting manifestation of severe combined immunodeficiency [15]. Under such circumstances, close monitoring of peripheral CD4 T lymphocyte count and serial examination of respiratory samples for Pneumocystis *jiroveci *might be helpful for earlier diagnosis and improving prognosis.

## Conclusion

The present case demonstrated the co-morbidity of PCP in a chemotherapy and radiotherapy-naïve, HIV-seronegative lung cancer patient with grave clinical outcome. Prompt diagnosis of PCP as a sequel of lung cancer can be difficult because of overlap in the clinicoradiological manifestations, and in the disturbed cellular immunological profile. Clinical and microbiological evaluations are indicated in those at high risk of cellular immunity deficiency, including subjects who undergo prolonged treatment with high dose corticosteroids.

## List of abbreviations

BAC = bronchioloalveolar carcinoma

CRP = C reactive protein

LDH = lactate dehydrogenase

PCP = Pneumocystis *jiroveci *pneumonia

## Competing interests

The author(s) declare that they have no competing interests.

## Authors' contributions

Drs C.C, X.ZH, Z.SQ, Z.NS were responsible for clinical management of the patient, and collection and interpretation of clinical data, Dr G.YY participated in the interpretation of histology and microbiology data, Dr Z.QS was in charge of the radiological manifestations of lung cancer and Pneumocystis *jiroveci *pneumonia. All participated in the discussion of the present case and contributed to the drafting of the manuscript.
